# The Relation Between Posture and Dimension of Tongue and Different Sagittal Skeletal Patterns and Vertical Skeletal Patterns in Class I and Class II Patients: A CBCT Study

**DOI:** 10.1155/ijod/5529949

**Published:** 2026-02-12

**Authors:** Soodeh Tahmasbi, Dena Bakhtiari, Kazem Dalaie, Yaser Safi, Fatemeh Eskandarloo

**Affiliations:** ^1^ Department of Orthodontics, School of Dentistry, Shahid Beheshti University of Medical Sciences, Tehran, Iran, sbmu.ac.ir; ^2^ School of Dentistry, Shahid Beheshti University of Medical Sciences, Tehran, Iran, sbmu.ac.ir; ^3^ Oral and Maxillofacial Radiology Department, School of Dentistry, Shahid Beheshti University of Medical Sciences, Tehran, Iran, sbmu.ac.ir; ^4^ Department of Orthodontics, School of Dentistry, Hamadan University of Medical Sciences, Hamadan, Iran, umsha.ac.ir

**Keywords:** CBCT, malocclusion, skeletal pattern, tongue posture, tongue volume

## Abstract

**Background:**

This study aimed to define the relation between posture and dimension of the tongue and different skeletal patterns in sagittal and vertical dimensions through cone beam computed tomography (CBCT).

**Method:**

CBCT images of 225 patients (149 females and 76 males) with a mean age of 33 years were included in this retrospective study. The sample was divided into three groups: class I (100 patients), class II (100 patients), and class III (25 patients). For each sample, tongue length (TGL) and tongue height (TGH) along with its position were evaluated. For statistical analysis, one‐way ANOVA, Tukey HSD, Welch’s *t*‐test, and Games–Howell test were performed. Also, to define the correlation between variables, Pearson and Spearman correlation coefficients were used.

**Results:**

The mean TGH was significantly greater in the class III group compared to class I (*p*‐value = 0.017) and class II groups (*p*‐value = 0.002). The mean TGL was significantly greater in the class II group compared to the class I group (*p*‐value = 0.008), without significant differences between other groups. There was no significant difference between vertical pattern groups in any of the tongue variables including TGL, TGH, mandibular length, and tongue position (*p*‐value > 0.05).

**Conclusion:**

The TGL was greater in class II patients, and the TGH was greater in class III patients. Also, class II patients had higher tongue posture compared to class I and III groups. Furthermore, vertical pattern had no significant influence on tongue position and dimension.

## 1. Introduction

Based on previous studies, it is known that the tongue’s dimensions and posture at rest have a crucial role in the etiology of malocclusion, orthodontic treatment planning, and occurrence of relapse after cessation of orthodontic treatments [1–3]. Tooth arches, which are aligned around the outer border of the tongue and surrounded by the muscles of lips and cheeks, are stabilized by the equilibrium of light and continuous forces by the tongue and circumoral muscles [4–6].

Considering the significant role of the tongue in tooth position, dental arch form, and occlusion, incontestably tongue posture and size should be considered during orthodontic and orthopedic treatment planning [7].

Previous studies have assessed the relationship between tongue characteristics and malocclusion patterns through different approaches, such as two‐dimensional (2D) radiographs (lateral cephalograms) [3, 8], three‐dimensional (3D) radiographs (cone beam computed tomography [CBCT]) [9], 3D ultrasound [10], or intraoral manometry [11] and have shown various results. Considering that CBCT provides more reliable and accurate results than lateral cephalograms for the evaluation of maxillofacial structures and is more accessible than others for orthodontists, it far outweighs the other methods in recent relevant studies [12].

Despite some studies claiming no significant relationship between tongue posture at rest and different skeletal malocclusions [13], other studies have shown distinct tongue posture and size between different sagittal [9, 14, 15] and vertical skeletal patterns [3, 16]. However, despite conducting numerous studies in this context through 3D assessments, there is still no consensus on the accurate relation between tongue characteristics and different malocclusions [17]. Accordingly, the aim of this study was the implementation of a more comprehensive study through a larger sample size and measurement of more 3D landmarks in CBCT to define the relation between posture and dimension of the tongue and different skeletal patterns. Also, in previous studies, most of the subjects were divided into general skeletal groups such as sagittal or vertical skeletal groups [13], but in the present study, sagittal class I and class II groups were divided into three vertical subgroups. The null hypothesis was considered as the absence of a relationship between them.

## 2. Materials and Methods

This study has been approved by the ethics committee with a number of IR.SBMU.DRC.REC.1400.062.

In this cross‐sectional study, the CBCT documents of patients referred from two oral and maxillofacial radiology centers from 2016 to 2021 were gathered and evaluated to enter the study based on the following eligibility criteria:

– Inclusion criteria: patients over 18 years old, with at least six permanent teeth at each quadrant without significant crowding, CBCT with a large field of view with maximum 80 kV, maximum 2 mA, 17 s exposure time, and 0.39 mm voxel size, with teeth in habitual occlusion relation and tongue posture at rest without complete contact with the palate.
– Exclusion criteria: any craniofacial or dental anomalies including missing or supernumerary teeth, history of trauma, history of previous orthodontic/orthopedic treatments, or craniofacial surgery especially involving tongue or facial muscles, under orthodontic/orthopedic treatments.

In the next step, after evaluating 609 documents, the radiographs matching eligibility criteria were divided into three groups in the context of the sagittal skeletal pattern (class I, II, and III) and three groups in the context of the vertical skeletal pattern (low angle, normal, and high angle) except class III group, which will be explained in later sections. Based on the following formula, the sample size was considered at least 30 patients for each group. But due to the lesser prevalence of class III malocclusion in the Iranian population, 25 samples were considered for the class III group and a total of 225 samples were evaluated in this study.

n=Z1−α2+Z1−β12ln1+r1−r2+3.

Each CBCT was reconstructed through Ondemand 3D software (Cybermed Co., Seoul, Korea, Version 1.0.10.6388).

Measurements were performed by a trained and blinded dentistry student (second author), and then to evaluate intra‐rater reliability rate, 20 randomly selected radiographs were remeasured by the same operator after 2 weeks and to evaluate inter‐rater reliability rate, and 20 randomly selected radiographs were remeasured by an orthodontist.

For each sample, the patient’s information including gender and age, skeletal pattern group type, and measurements of skeletal relations, tongue, and upper pharyngeal airway were recorded as follows.

### 2.1. Definition of the Skeletal Landmarks

For each sample, to prevent superimposition, in the coronal view, the right half of the CBCT was omitted, and then the following landmarks were defined to be used for skeletal and tongue analyses (Jarabak uses S/Go/N/Me, mandibular plane angle uses S/N/Go/Me, ANB uses A/N/B, Wits uses A/B, inclination angle uses Se/N/ANS/PNS, tongue length (TGL) uses EB/TT, and mandibular length uses Go/Gn/Pog/Me).

• N (Nasion): the most anterior point of the frontonasal suture
• S: the geometric center of Sella turcica
• Se: the midpoint of the entrance of Sella turcica
• ANS: the tip of the anterior nasal spine
• PNS: the tip of the posterior spine of the palatine bone
• Point A: the innermost point on the contour of the premaxilla between the anterior nasal spine and the incisor tooth
• Point B: the innermost point on the contour of the mandible between the incisor tooth and chin
• Go: the midpoint of the contour connecting the ramus and body of the mandible
• Pog: the most anterior point on the mandibular symphysis
• Gn: the most inferior and anterior point on the mandibular symphysis
• Me: the most inferior point on the mandibular symphysis
• EB: base of epiglottis
• TT: the most anterior point of the tongue tip

### 2.2. Skeletal Analysis

The following analyses were performed:

• Jarabak index: the ratio of posterior facial height (S‐Go) to anterior facial height (N‐Me) in the midsagittal plane
  Normal: 62%–65%, low angle: >65%, and high angle: <62%
• Mandibular plane angle (SN‐Go‐Me): the angle between Sella‐Nasion plane and mandibular plane (Go to Me)
  Normal: 28.5–39.5°, low angle: <28.5°, and high angle: >39.5°
• ANB: the angle between point A, Nasion, and point B in the midsagittal plane
  Class I: 0–4°, class II: >4°, and class III: <0°
• Wits analysis: the linear distance between the projection of point A and point B on the functional occlusal plane, which is formed by bisecting the intercuspation of first premolars and first molars
  Class I: −1–1 mm, class II: >1 mm, and class III: <−1 mm
• Inclination angle: the angle between the perpendicular line dropped from the Se‐N plane at N point to the palatal plane (ANS‐PNS)e
  Normal: 81.1–88.9°, retro inclination: <81.1°, and ante inclination: >88.9°

Based on the mentioned measurements, the samples were divided into 7 groups: (1) class I—normal growth pattern (33 samples), (2) class I—low angle (37 samples), (3) class I—high angle (30 samples), (4) class II—normal growth pattern (33 samples), (5) class II—low angle (36 samples), (6) class II—high angle (31 samples), and (7) class III (25 samples).

### 2.3. Tongue Analysis

The following analyses were performed (Figure 1):

• TGL: the linear distance between EB and TT in the midsagittal plane.
• Tongue height (TGH): the linear distance on the perpendicular line from the middle of TGL to the posterior surface of the tongue
• Mandibular length (Go‐Gn): the linear distance between Go and the most anterior point of the mandibular base, defined by a perpendicular line from Pog to the mandibular plane (Go‐Me)
• Tongue position: after drawing a horizontal line from the incisal edge of the lower central incisor to the cervical distal third of the lower second molar, considering the cervical area of the second molar as the center, angles were drawn at 30°, 60°, 90°, 120°, and 150°. On the designated lines, the distance between the center and the tongue contour was considered as D1 to D5, respectively, and the distance between the tongue contour and palate was considered as D1′ to D5′, respectively.

Figure 1Measurement of (a) TGL, (b) TGH, and (c) tongue position including D1 to D5 and D1’ to D5’.

**Figure:**
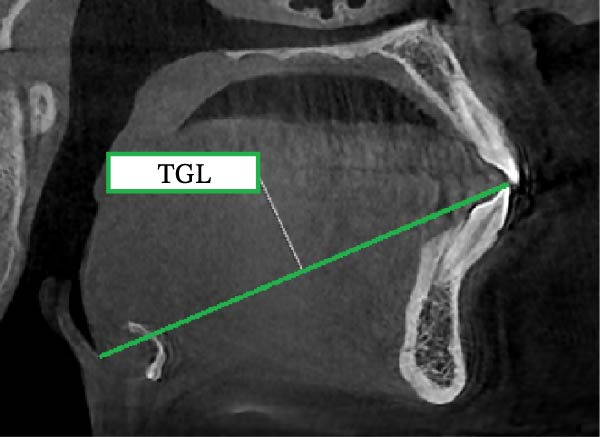
(a)

**Figure:**
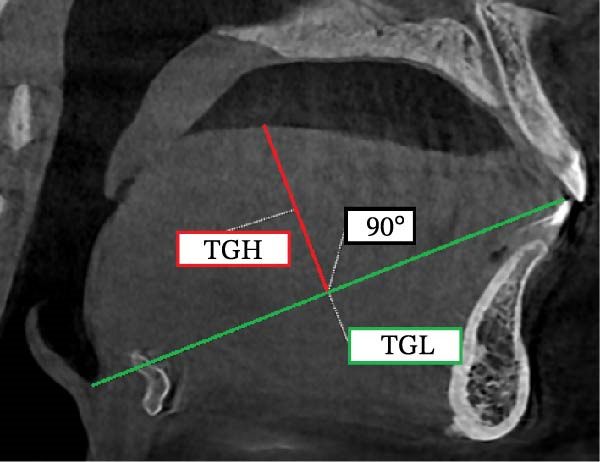
(b)

**Figure:**
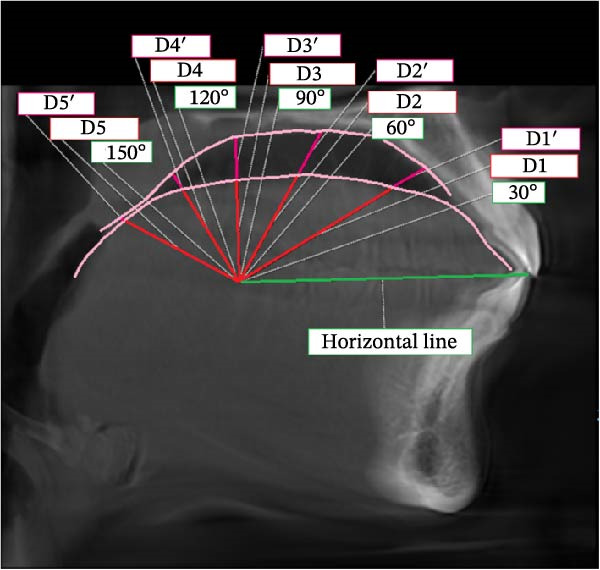
(c)

### 2.4. Data Analysis

Finally, all data were recorded and gathered in Excel software, and the data analysis was conducted through SPSS software version 20.0. The Paired *t*‐test and intraclass correlation coefficient (ICC) were used to define inter‐rater and intra‐rater reliability rates. The Kolmogorov–Smirnov test was performed to evaluate the normal distribution of resultant data. For data with a normal distribution, the Pearson correlation coefficient and for data without a normal distribution, the Spearman correlation coefficient was used. After performing Levene’s test to assess the equality of variances between groups, in the case of equality of variances, the one‐way ANOVA and Tukey HSD tests were utilized, and if the variances were not equal, Welch’s *t*‐test and Games–Howell test were utilized. Also, the two‐way ANOVA was performed to evaluate the interaction of sagittal and vertical measurements between groups. The significance level was considered as 0.05.

## 3. Results

In this study, CBCT images of 225 patients were evaluated, including 149 females (66.12%) and 76 males (33.8%). The mean age was 33.06 ± 10.77 years (min: 18 and max: 77).

Table 1 shows group sizes and vertical subgroup distributions.

**Table 1:** Group sizes and vertical subgroup distributions.

Sagittal skeletal pattern	Vertical skeletal pattern	Number of samples
Class I	Normal	33
	High angle	30
	Low angle	37
Class II	Normal	33
	High angle	31
	Low angle	36
Class III	—	25

The ICC for intra‐rater reliability across all tongue variables in 20 random patients was 1 with 94% CI (0.99–1). Also, the mean difference between the two measurements was 0.05 (*p*‐value = 0.410). So, the difference between the two measurements by the same operator was insignificant.

The ICC for inter‐rater reliability across all tongue variables in 20 random patients was 0.998 with 94% CI (0.997–1). Also, the mean difference between the two measurements was 0.01 (*p*‐value = 0.882). So, the difference between the two measurements by the two different operators was insignificant.

Based on the Kolmogorov–Smirnov test, the data distribution of most of the variables in class I, II, and III groups was normal or approximately normal (*p*‐value ≥ 0.05) except D1′, D2′, D4′, and D5′ in class I, II, and III groups; D2 and D3 in class I group; D3′ in class I and II groups; and TGL in class II group (for these data, the Spearman correlation coefficient was used). So, parametric tests were utilized for later analyses.

**Figure 2:**
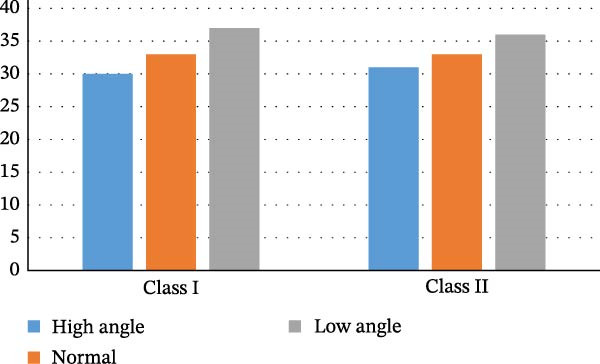
Distribution of vertical patterns in class I and II groups.

As mentioned previously, due to the lesser prevalence of class III malocclusion and smaller sample size, vertical pattern classification was not executed in this group. So, Figure 2 shows the distribution of vertical pattern distribution in each group of class I and II. In both groups, the highest prevalence belonged to the low‐angle group, followed by normal and high‐angle groups.

Table 2 shows the descriptive study of tongue variables in all seven groups (both sagittal and vertical pattern classifications).

**Table 2:** Descriptive study of tongue variables (in mm) in sagittal and vertical pattern classifications.

Groups									
Variables	Class I—high angle	Class I—normal	Class I—low angle	Class I—mean	Class II—high angle	Class II—normal	Class II—low angle	Class II—mean	Class III
TGH	35.6 ± 4.38	36.24 ± 4.38	34.87 ± 4.90	35.54 ± 4.34	34.24 ± 3.58	35.89 ± 4.36	36.49 ± 4.81	35.6 ± 4.37	38.26 ± 4.72
TGL	73.21 ± 5.02	74.0 ± 6.56	75.32 ± 7.47	75.46 ± 6.51	76.07 ± 5.72	76.58 ±6.69	75.89 ± 7.1	76.18 ± 6.5	75.26 ± 6.1
D1	29.33 ± 6.01	30.66 ± 4.14	30.79 ± 5.47	30.28 ± 5.24	31.31 ± 6.77	32.59 ± 5.14	33.57 ± 4.87	32.55 ± 5.63	30.31 ± 4.42
D1’	3.84 ± 5.15	2.62 ± 3.31	3.44 ± 4.17	3.29 ± 4.24	4.4 ± 5.45	3.02 ± 4.0	2.42 ± 3.15	3.23 ± 4.28	3.67 ± 3.78
D2	23.75 ± 5.4	24.83 ± 3.84	25.21 ± 9.56	25.37 ± 6.85	23.39 ± 5.34	25.46 ± 4.9	25.63 ± 4.62	25.88 ± 4.99	24.46 ± 4.74
D2’	4.2± 5.02	3.2 ± 3.88	3.8 ± 4.18	3.72 ± 4.33	4.05 ± 4.93	2.60 ± 3.32	2.53 ± 2.6	3.03 ± 3.71	3.74 ± 4.26
D3	20.41 ± 5.25	21.50 ± 3.54	22.07 ± 10.23	21.38 ± 7.12	19.05 ± 4.34	20.81 ± 4.21	21.62 ± 4.17	21.56 ± 4.33	20.61 ± 4.88
D3’	3.9 ± 5.04	2.44 ± 3.24	3.36 ± 4.07	3.22 ± 4.15	2.81 ± 3.67	1.73 ± 2.01	1.84 ± 2.11	2.1 ± 2.68	3.39 ± 3.87
D4	18.77 ± 4.11	19.79 ± 3.71	19.61 ± 4.66	22.42 ± 4.18	17.06 ± 3.44	18.01 ± 3.90	18.89 ± 3.48	23.03 ± 3.65	21.19 ± 4.84
D4’	1.58 ± 2.26	1.33 ± 2.45	1.83 ± 2.87	1.18 ± 2.55	1.26 ± 1.37	0.84 ± 0.89	0.77 ± 1.03	0.93 ± 1.12	1.34 ± 1.88
D5	19.9 ± 4.58	20.95 ± 4.09	20.49 ± 4.46	22.46 ± 4.35	18.39 ± 3.75	18.41 ± 4.12	19.63 ± 3.69	23.65 ± 4.29	21.57 ± 4.33
D5’	1.0 ± 1.5	1.43 ± 1.91	1.59 ± 1.97	1.36 ± 1.82	0.77 ± 1.1	0.8 ± 1.1	0.71 ± 0.9	0.75 ± 1.02	1.44 ± 1.61
Go‐Gn	73.5 ± 5.04	73.87 ± 4.59	73.4 ± 4.79	73.64 ± 4.74	70.78 ± 4.10	71.25 ± 4.68	72.74 ± 6.43	71.64 ± 5.24	77.93 ± 6.12

### 3.1. Comparison of the Tongue Variables Between Sagittal Pattern Groups

The result of Tukey HSD and Games–Howell tests is shown in Table 3.

**Table 3:** The result of Tukey HSD and Games–Howell tests.

Dependent variables	Sagittal skeletal pattern	Sagittal skeletal pattern	Mean difference	*p*‐Value
TGH	Class I	Class II	−0.05520	0.996
		Class III	−2.71770	0.017
	Class II	Class III	−2.66250	0.020
TGL	Class I	Class II	−2.26650	0.008
		Class III	−0.02660	1.000
	Class II	Class III	2.23990	0.149
D4	Class I	Class II	1.38730	0.042
		Class III	1.76950	0.125
	Class II	Class III	−3.15680	0.002
D4’	Class I	Class II	0.65610	0.052
		Class III	−0.24750	0.850
	Class II	Class III	−0.40860	0.560
D5	Class I	Class II	1.81010	0.010
		Class III	0.11280	0.485
	Class II	Class III	−2.92290	0.008
D5’	Class I	Class II	0.60890	0.011
		Class III	−0.32720	0.655
	Class II	Class III	−0.28170	0.688

Based on the Tukey HSD test, the mean of TGH was significantly greater in the class III group compared to class I (*p*‐value = 0.017) and class II groups (*p*‐value = 0.002). The mean of TGL was significantly greater in the class II group compared to the class I group (*p*‐value = 0.008), without significant differences between other groups. The mean of D4 and D5 was significantly greater in the class II group compared to class I (*p*‐value = 0.042 and 0.01, respectively) and class III (*p*‐value = 0.002 and 0.008, respectively) groups.

Based on the Games–Howell test, the mean of D5′ was significantly greater in class I compared to the class II group (*p*‐value = 0.011), without significant difference between other groups.

No significant difference was observed between groups in other tongue variables.

### 3.2. Comparison of the Tongue Variables Between Sagittal and Vertical Pattern Groups

Based on two‐way ANOVA analysis, there was no significant difference between vertical pattern groups in none of the tongue variables including TGL, TGH, Go‐Gn, and tongue position parameters (D1 to D5 and D1′ to D5′) (*p*‐value > 0.05). Also, there was no significant interaction between vertical and sagittal groups in none of the tongue variables (*p*‐value > 0.05).

### 3.3. The Relation Between the Tongue Variables in Sagittal Pattern Groups

The correlation coefficient between Go‐Gn and other tongue variables is shown in Table 4. As shown, there was only a significant correlation between Go‐Gn and TGH in class II, TGL in class II, D1 in all groups, D1′ in class III (negative correlation), D2 in class II, D3 and D4 in class II and III, and D5 in all groups.

**Table 4:** Correlation of tongue variables between sagittal pattern groups.

Variable	Go‐Gn					
	Class I		Class II		Class III	
	Correlation coefficient	*p*‐Value	Correlation coefficient	*p*‐Value	Correlation coefficient	*p*‐Value
TGH	0.155	0.124	0.253	0.011 ^∗^	0.376	0.064
TGL	−0.083	0.410	0.261	0.009 ^∗^	−0.319	0.120
D1	0.303	0.002 ^∗^	0.364	0.001 ^∗^	0.470	0.018 ^∗^
D1’	0.075	0.456	−0.130	0.196	−0.433	0.031 ^∗^
D2	−0.007	0.947	0.305	0.002 ^∗^	0.108	0.609
D2’	0.079	0.435	−0.053	0.601	−0.065	0.757
D3	0.087	0.389	0.306	0.002 ^∗^	0.401	0.047 ^∗^
D3’	0.101	0.317	−0.086	0.396	0.172	0.411
D4	0.194	0.054	0.320	0.001 ^∗^	0.562	0.003 ^∗^
D4’	−0.004	0.972	−0.100	0.322	0.105	0.616
D5	0.249	0.012	0.288	0.004 ^∗^	0.506	0.010 ^∗^
D5’	0.063	0.534	−0.083	0.414	−0.039	0.855

^∗^Indicates statistical significance at 0.05.

## 4. Discussion

In the present study, the relation between tongue features such as length, height, and position and different sagittal and vertical skeletal patterns was evaluated through CBCT of 225 patients. The samples were divided into three sagittal pattern groups based on measurement of ANB angle and Witts number in the midsagittal plane, and subsequently, the class I and II groups were divided into three vertical pattern groups based on measurements of Jarabak index, mandibular plane angle, and inclination angle.

Evaluating tongue posture and dimension is crucial during orthodontic treatment planning to improve the treatment results and stability. The tongue, as one of the prior components of intraoral equilibrium, plays an important role in establishing the arch form and occlusal relationship [18–20]. So, any changes in tongue position or dimension will change the equilibrium and cause instability of treatment results [8].

For this study, CBCT was utilized [21], as it is clearly shown to be more accurate and reliable than lateral cephalograms [22, 23]. Also, a preliminary study by Halim et al. [24] confirmed that evaluating tongue volume through chosen landmarks in CBCTs is reproducible and reliable. Considering that this study was retrospective, the used CBCTs were taken previously for other diagnostic or therapeutic purposes, so none of the samples was disposed to abundant exposure. Another advantage of CBCT is that despite cephalograms, the definition of landmarks is not affected by the patient’s head orientation [25]. In this study, all measurements were conducted by a trained dentistry student, who was blind to the patient’s information, with high inter‐reliability and intrareliability rates.

Previous similar studies had a lower sample size [26], but in the present study, 225 CBCTs were assessed, as well as dividing the samples into more precise groups including both sagittal and vertical skeletal measurements. Previous studies divided the patients into only sagittal or vertical groups [13]. However, in this study, each of the class I and II groups was also classified into three vertical groups to evaluate the interaction between two planes.

According to the results of the present study, the length of the tongue was significantly greater in the class II group in comparison to class I. There was no significant difference between different vertical patterns in either group in the context of TGL. Similarly, another CBCT study by Grover et al. [27] confirmed that tongue volume had no significant difference between different vertical skeletal patterns. In another study, Chen et al. [9] concluded that there was no significant difference between class I and II patients in the field of TGL, but the TGL was significantly greater in the class I low‐angle group, contrary to the results of the present study. Although similar to the present study, they concluded that tongue features are not affected by vertical skeletal pattern in class II patients.

This study also revealed that TGH is significantly greater in class III patients in comparison to class I and II groups. Although, in class II patients, posterior tongue posture (D4 and D5) was significantly higher in comparison to other groups. Tongue position in anterior and middle regions had no significant difference between groups. On the other hand, a greater distance between the dorsal surface of the tongue and palate at the posterior region (D4′ and D5′) was observed in the class I group in comparison to only the class II group, resulting in a lower posterior posture of tongue in class I skeletal pattern. Contrary to these studies, Chen and colleagues [9] showed that tongue posture is lower in the class II group in comparison to class I, similar to the study by Iwasaki et al. [26]. The difference between the results of two studies might be due to different measurement methods for tongue posture. On the other hand, Primozic et al. [15] claimed lower tongue posture in class III patients in comparison to class I pattern, similar to the results of the study by Fatima and Fida [13] and Ha et al. [28]. In another study, Sun et al. [8] concluded that lower tongue position is linked to skeletal class III malocclusion, which is consistent with the results of the present study. Although tongue posture had no significant difference between different vertical patterns in the present study, previous studies [3, 16] showed higher tongue posture in long face patients.

Considering that the present study was retrospective, it was not possible to conduct different measurement methods on CBCTs. Also, the age range was very wide along with a lower number of class III patients, which could be improved by increasing sample size. The position of the hyoid bone was not evaluated in this study. Consequently, it is suggested to conduct studies with a greater sample size with more class III samples and also to use a more reliable approach to assess the tongue posture in CBCT.

The lack of measurement of tongue volume was one of the limitations of this study. It also seems that well‐designed studies are needed to investigate the clinical effects of tongue posture and dimensions on various issues related to diagnosis and treatment and relapse risk.

## 5. Conclusion

In this retrospective study, based on the evaluation of CBCTs, it was concluded that TGL was greater in class II patients, and TGH was greater in class III patients. Also, class II patients had higher tongue posture in comparison to class I and III groups. Furthermore, the vertical pattern had no significant influence on any of the features of tongue position and dimension.

## Funding

This research was financially supported by the Dental Research Center, Research Institute of Dental Sciences, School of Dentistry, Shahid Beheshti University of Medical Science, Tehran, Iran.

## Conflicts of Interest

The authors declare no conflicts of interest.

## Data Availability

The datasets used and/or analyzed during the current study are available from the corresponding author upon reasonable request. Also, the datasets supporting the conclusions of this article are included within the article.
